# Economic Crisis: A Factor for the Delayed Diagnosis of Breast Cancer

**DOI:** 10.3390/ijerph18083998

**Published:** 2021-04-10

**Authors:** Iasmina Petrovici, Mihaela Ionica, Octavian C. Neagoe

**Affiliations:** 1 Department of Philosophy and Communication Sciences, Faculty of Political Sciences, Philosophy and Communication Sciences, West University of Timişoara, Vasile Parvan Boulevard No. 4, 300223 Timișoara, Romania; iasmina.petrovici@e-uvt.ro; 2 Second Clinic of General Surgery and Surgical Oncology, Emergency Clinical Municipal Hospital Timișoara, Gheorghe Dima Street No. 5, 300079 Timișoara, Romania; neagoe.octavian@umft.ro; 3 Department of Pathophysiology-Functional Sciences, “Victor Babeş” University of Medicine and Pharmacy Timișoara, Eftimie Murgu Square No. 2, 300041 Timișoara, Romania; 4 Second Discipline of Surgical Semiology, First Department of Surgery, ”Victor Babeș” University of Medicine and Pharmacy Timișoara, Eftimie Murgu Square No. 2, 300041 Timișoara, Romania

**Keywords:** breast cancer, public health, risk factor, economic crisis

## Abstract

Considering the constant increase in breast cancer patients, identifying factors that influence the moment of diagnosis is essential for optimizing therapeutic management and associated cost. The purpose of the study is to evaluate the impact of the economic crisis on the moment of a breast cancer diagnosis. This retrospective observational study analyzed a cohort of 4929 patients diagnosed with breast cancer over the course of 19 years in the Western region of Romania. The time interval was divided based on the onset of the economic crisis into 3 periods: pre-crisis (2001–2006), crisis (2007–2012), and post-crisis (2013–2019). The disease stage at the moment of diagnosis was considered either early (stages 0, I, II) or advanced (stages III, IV). Although recording a similar mean number of patients diagnosed per year during the pre- and crisis periods, a significantly higher percentage of patients were diagnosed with late-stage breast cancer during the economic crisis period compared to the previous interval (46.9% vs. 56.3%, *p* < 0.01). This difference was further accentuated when accounting for environmental setting, with 65.2% of patients from a rural setting being diagnosed with advanced disease during the crisis interval. An overall improvement of 12% in early-stage breast cancer diagnosis was recorded in the post-crisis period (55.7%, *p* < 0.001). The findings of this study support periods of economic instability as potential factors for a delay in breast cancer diagnosis and highlight the need for the development of specific strategies aimed at reducing cancer healthcare and associated financial burden in times of economic crisis.

## 1. Introduction

Breast cancer is among the most common forms of cancer diagnosed in women and although survival rates can reach up to 90% at 5 years, this disease represents a leading cause of death through cancer, particularly in low- and middle-income countries [1]. Furthermore, both medical and sociological surveillance data report a continuous rise in worldwide incidence [1,2,3,4]. This trend in breast cancer evolution suggests that associated healthcare costs can be expected to increase in the near future, especially in the case of patients with advanced stages at diagnosis. Ensuring timely diagnosis is essential for both long-term survival and reducing medical care costs [5,6,7]. A shorter time to an earlier diagnosis has been shown to associate a more favorable outcome [5], an aspect supported by survival rates of breast cancer patients. Survival data for breast cancer has consistently been shown to be directly proportional to the stage at diagnosis. The Surveillance, Epidemiology, and End Results (SEER) Program has reported that 5-year survival rates for localized disease can reach 99%, recording a steady decrease with breast cancer progression. Regionally spread breast cancer records a 5-year survival of 86%, while only 28% of patients with distant disease are alive at 5 years from the moment of diagnosis [6]. Another important aspect correlated to timely diagnosis is represented by the financial burden required to treat these patients. In a comprehensive review by Sun et al. on the costs of breast cancer treatment, stage of disease was a significant factor for healthcare cost increase. Compared to localized breast cancer forms, costs increased by 41% and 165% for patients with regional and distant diseases, respectively [7]. This financial aspect becomes even more important in the context of economic instability, such as seen during an economic crisis. 

Without going into detail, at the origin of the economic crisis is the worldwide financial crisis [8,9,10,11] and although certain causes can be identified in the previous years, specialists consider that it has started in February 2007, with obvious consequences being more evident in July and August 2007 [12,13,14]. The financial crisis has manifested itself differently in the affected countries and, depending on how strict fiscal austerity measures have been implemented, serious consequences have been observed on both vulnerable populations, as well as on the health system [9,15,16,17]. Consequences of financial instability were observed quite quickly in Romania [13], being confronted with an ample economic crisis, spread over a period of six years. Although Romania, like other EU countries, has benefited from international “multilateral financial assistance” since the beginning of the crisis [15], corrective measures, fiscal and budgetary policies have been implemented later than in other countries, namely only in 2009. Moreover, due to the fact that some of these measures proved unviable (except for those aimed at reducing public spending), socioeconomic conditions continued to deteriorate and the crisis lasted until 2012. Additionally, austerity measures and budgetary restrictions adopted by the Romanian government in 2009 (the most notable ones being the reduction of salaries, dismissals, and blocking of employment in the public sector) have led to an exacerbation of the crisis and a drastic decrease in consumption [13,15,18], the peak of the crisis being considered between 2009 and the first half of 2012.

Cancer mortality, in general, has been shown to increase during periods of economic instability [19,20], the crisis of 2007–2008 is estimated to have generated an added 260,000 cancer-related deaths in The Organization for Economic Cooperation and Development alone [21]. Financial disparity has a significant impact on health inequalities, a condition that has both short and long-term deleterious effects on public health, particularly in at-risk population groups [19,20,22]. Maynou et al. have shown in an analysis of the effect of economic crisis on overall mortality and cause-specific mortality in the European Union, that implementation of austerity measures increased health disparities secondary to socioeconomic inequalities [20]. As previously mentioned, one of the effects of the economic crisis was reflected in an increase in the unemployment rate, with a direct consequence on individual income level. Merino-Ventosa et al. have shown that during the economic crisis income-related inequalities have determined a decrease in cervical cancer screening for individuals with a lower income, despite an increase in screening capacity over time [22]. On a more general note, all-cause cancer mortality rates were significantly increased and correlated to unemployment rate in a longitudinal analysis of 75 countries worldwide. The same study highlights that universal health coverage may have a protective effect against the consequences of unemployment [21]. However, the aspect of universal health coverage is extremely variable, depending on local health policies, an aspect that was not controlled for in the analysis. In the same line, although Romanian health policies provide universal health coverage, this aspect is conditioned by employment status; loss of employment resulting in most cases in the loss of this universal coverage. Specific analysis of the economic crisis impact on breast cancer diagnostic and care has is scarce in the available literature, particularly for Eastern European countries, such as Romania. 

The present study uses an interdisciplinary approach to explore the impact of the economic crisis on breast cancer prognosis. We hypothesize that during this period of economic instability a change in population behavior could be observed with regard to the addressability to medical services, determining a delay in the initial moment of breast cancer diagnosis, reflected by a higher proportion of patients with late-stage disease at presentation. To this end, the influence of the economic factor on the moment of breast cancer diagnosis was evaluated in the population of the Western region of Romania, based on a comparative analysis of data recorded before, during, and after the economic crisis of 2007. This study is of particular relevance in the current worldwide situation, as the COVID-19 pandemic has determined serious economic instability and distress, doubled by a medical crisis due to the consumption of vital healthcare resources. 

## 2. Materials and Methods

For the purpose of this study, a retrospective observational analysis of data was carried out on a cohort of patients diagnosed with breast cancer in the Western region of Romania. Data were extracted retrospectively from the Western Oncologic Registry and the Medical Oncology Clinic of the Emergency Clinical Municipal Hospital Timisoara. Collected data spanned across 19 years, between 2001 and 2019. Inclusion criteria were represented by the diagnosis of breast cancer, diagnosed in Timis County between 2001 and 2019, age over 18 years, all genders, all stages. Exclusion criteria comprised patients with the age below 18 years, and patients with missing data regarding age, gender, stage at diagnosis, or environmental setting. A cohort comprising of 4929 patients diagnosed with breast cancer was obtained following the selection of patients. 

For the evaluation of the influence of the economic crisis on the stage of disease at the moment of breast cancer diagnosis, the study interval was divided into 3 representative periods for the economic crisis: pre-crisis (2001–2006), crisis (2007–2012) and post-crisis (2013–2019). Disease severity at the moment of diagnosis was considered a dichotomous variable and classified as either early or advanced stage. Early disease comprised stages 0, I, and II, while advanced breast cancer was considered stages III and IV. 

Statistical analysis was performed using SPSS software for Windows version 21. Descriptive analysis was performed for: (i) continuous data, represented by the variable ‘age’, being presented as mean range and standard deviation; and (ii) categorical data, represented by the period of diagnosis (pre-, crisis, and post-crisis), disease stage (early vs. advanced) and environmental setting (urban vs. rural), being expressed as proportions and/or frequencies. Comparison of mean values between groups for the continuous variable was performed through one- and two-way ANOVA analysis. The correlation of categorical variables was assessed through Pearson’s chi-squared test. Cramer’s V analysis was used to assess the effect size of the Pearson correlation. A *p*-value of <0.05 was considered statistically significant.

## 3. Results

From the total of 4929 patients diagnosed with breast cancer, the vast majority was represented by female patients (97.3%). This gender distribution was similar across all three periods evaluated in this study, without a significant difference being recorded between these intervals. The mean age recorded for the whole group was 51.2 ± 17.4 years, with an observed minimum value of 21 years and a maximum value of 91 years, respectively. Analysis of mean age differences between the pre-crisis, crisis, and post-crisis periods showed no clinically significant differences. Similarly, within each time-interval group (pre-, crisis, and post-crisis) no significant mean age differences were observed between early versus late-stage patients, nor between patients from a rural or urban setting. 

The mean number of patients diagnosed during the economic crisis and pre-crisis periods recorded similar values; although fewer patients were observed during the crisis period (Table 1), no statistical significance was found. A significantly higher number of patients was recorded during the post-crisis period than in the previous two intervals (*p* < 0.001), representing almost half of the study group (47.0%).

Following the distribution of patients by disease stage with regard to the year of diagnosis, an interesting pattern emerged. A statistically significant drop of 9.4% was recorded during the economic crisis period in patients with early-stage breast cancer, thus determining a raise in advanced disease cases from 46.9% in the pre-crisis period to 56.3% (*p* < 0.001). A reversed situation was recorded during the post-crisis period with a 12% reduction of late-stage patients being diagnosed (*p* < 0.001), with more than half of patients being treated for early-stage breast cancer (Table 2). 

This difference between early and advanced stages at diagnosis becomes even more evident when analyzing the urban/rural distribution of the patients across the three time intervals. In the pre-crisis period, more than half of patients living in an urban setting were diagnosed with breast cancer at an early stage (56.4%). For the same interval, the distribution of patients from the rural areas recorded slightly more patients with advanced stages (51.2%). Although the results were statistically significant, only a small effect was observed. During the crisis period, in the rural environment diagnosis at an advanced stage was significantly higher compared to an early diagnosis (*p* < 0.001), late-stage breast cancer being present in 65.2% of cases, thus recording also a significant increase by comparison to the previous period for patients in a rural setting (*p* < 0.01). However, during the economic crisis period patients from an urban setting recorded a similar distribution between early and late-stage at diagnosis, with no significant difference within the time interval. However, a significant decrease in early stages between the pre-crisis and crisis periods was observed for patients from an urban environment (*p* < 0.01), with a moderate effect size. The post-crisis period recorded a significant improvement in the moment of breast cancer diagnosis, early detection being observed in 59.8% and 49.1% of patients from the urban and rural areas, respectively (Table 3). Within the post-crisis period, the observed differences in the moment of diagnosis were statistically significant only for patients from an urban setting (*p* < 0.01). However, significant differences were recorded for both settings when comparing to the previous time interval–crisis period (*p* < 0.001), with a moderate effect size. 

## 4. Discussion

The present study focused on the influence of the economic crisis on the moment of diagnosis of breast cancer. We considered this interdisciplinary approach, given that there are currently few studies on how the crisis has influenced health policies in Romania, in particular, on how it influenced the diagnosis, treatment, and prognosis of breast cancer in the western region of the country. Although the World Health Organization has set up a working body to assess the sustainability of EU countries’ health systems facing economic crises, limited data is available on how the crisis has influenced health policies in Romania.

As stated previously the start of the economic crisis worldwide is considered February 2007 [12,13,14]. The implementation of the Fair Minimum Wage Act of 2007, the decline of investors’ confidence in secured mortgages, the liquidity crisis, the collapse of the stock market, the bankruptcy of a large number of banks, insurance companies, and creditors, the increase of public debts have led to a systemic financial crisis worldwide since July 2007 [23,24]. The financial crisis has manifested itself differently in the affected countries and depending on how strict fiscal austerity measures have been implemented serious consequences have been observed on both vulnerable populations, as well as on the health system [9,15]. In Romania, the consequences of financial instability became apparent rapidly [13], and spanned over a six-year period. By adopting the Fair Minimum Wage Act, in parallel with the increase in budget expenditures (immediately after the parliamentary elections of December 2008), in Romania, the financial and economic instability worsened in the following year. Furthermore, while the unemployment level increased and salaries were reduced, the level of prices remained unchanged, thus generating a dramatic decrease in consumption, along with a decrease in access to various services, including healthcare. Per capita, public expenditure on healthcare has decreased between 2007 and 2012 [15]. During the crisis, in Romania an increase in financial disparities was observed, manifested, mainly, in the large inequalities between the salary of the employees from the public sector and those from the private sector; similarly, between the population categories that used the income to speculate on financial markets and the population categories that extended loans in order to maintain a decent standard of living. It was only at the end of 2012 that the new government implemented sustainable policies in the financial system, a series of restrictive policy measures and crisis resolution tools that contributed to the increase in consumption and, thus, to a slight economic recovery.

The interdisciplinary approach of our study is based on the health model of Marc Lalonde [25,26,27]. In this model, it is argued that the health of a population is determined and influenced by biological factors (age and genetic inheritance), environmental factors, the healthcare system, and behavior (lifestyle) [26,28,29,30]. The infrastructure and organization within each state of public and private healthcare systems have a considerable impact on the population health status, healthcare infrastructure being directly correlated to the economic status of the respective country [31,32,33]. Thus, the economic power of a country greatly influences choosing a more efficient medical system, an aspect that can be reflected by optimal population health scores and higher life expectancy [31,32]. Countries where socioeconomic inequalities are prevalent, such as marked income disproportion (more individuals with low income compared to individuals with high income) are faced with more problems in ensuring a satisfactory level of public health. Such economic inequalities can have a predictive role. As studies show, economic disparities present during adolescence can determine a potential health issue in adult life. Moreover, by evaluating the correlation of both subjective and objective measures of socioeconomic status to adolescent health, Ahlborg et al. show that subjective measures are a strong and more reliable predictor of health status [34]. An important aspect is thus highlighted, namely that the individuals’ perception regarding their socioeconomic status can significantly influence personal health. 

Applying the health model of Lalonde it can be observed that the economic factor influences also the demographic characteristics of breast cancer. In developing countries, low economic status is associated with a poorly developed medical infrastructure, decreased access to quality medical services, and a lack of screening programs, with a consequent increase in mortality [35,36,37]. The increased life expectancy has determined a longer exposure to risk factors, thus recording a raise in oncologic disease among women, breast cancer accounting for 25.1% of all cancers according to GLOBOCAN data [1,4]. It is estimated that in the next two decades the number of breast cancer patients will amount to 22 million new cases worldwide [1,4]. In Romania, a national screening program for breast cancer has existed since 2012, however, the application of these timely diagnostic procedures is extremely heterogenous and ineffective, with only a small percentage of the population benefiting from early diagnosis. According to Eurostat data, in 2015 only 0.2% of females from Romania with ages between 50–69 years participated in screening programs [3].

The incidence of breast cancer increases significantly in developing countries from South America, Africa, and Asia, where the economic factor is fluctuant, studies showing that early detection of the disease has a major impact on prognosis and therapy [2]. If in developed countries more than 50% of patients benefit from screening programs, less developed countries are often lacking such programs, with a consequently decreased rate of survival. In these countries not only, the sociocultural factor has an important role (low population health education), but also the low socioeconomic status by determining low and/or late population addressability to medical services [2,38]. 

An economic crisis represents a moment of fall in a country, with factors of the Lalonde model being radically changed and thus greatly influencing population health. In general, regardless of the determining causes, a period of economic recession is characterized by several factors such as increased unemployment rate, inflation, income inequality, decreased production or-for capitalist countries-overproduction correlated with an acute decrease in economic activity, diminished demand, financial crisis, public indebtments. Recent studies have shown that the economic crisis has a significant impact on the management of cancer patients, with the unemployment rate being correlated with a poor prognosis [19,39]. Raises in unemployment have been shown to significantly increase breast cancer mortality across the European Union, regardless of healthcare infrastructure [19]. Furthermore, austerity measures required to limit the financial effects of the crisis have been demonstrated to significantly impact health inequalities [20]. The presence of such inequalities represents a basis for a delayed cancer diagnosis, particularly in individuals with a low education level, without medical insurance coverage, or with low income [22]. These differences in health inequalities during periods of economic crisis may be more prevalent in low- and middle-income countries, where several factors may contribute to accentuate the negative effects of financial disparities [20,22]. As seen in the present study the economic factor determines a significant delay in the moment of diagnosis, recording a more advanced breast cancer stage. The moment of diagnosis is an essential aspect of the long-term prognosis. Ten-year survival rates can reach up to 95% for patients with stages 0 and I, but decrease significantly with stage progression, with rates between 10–60% for stage IIIA, less than 30–35% for stages IIIB–C, and only 0.5% for patients with stage IV. In Romania, the previously mentioned inequalities are further accentuated by the differences between the urban and rural settings. Despite recording a similar number of patients in both the pre- and crisis periods, a significant increase in advanced breast cancer cases was observed during the crisis period, particularly in the rural setting. The prognosis of these patients may be further compromised as economic instability and increases in unemployment rates have been shown to make patients less likely to attend follow-up protocol [19], thus potentially delaying the diagnosis of recurrences or disease progression. The results obtained from the present analysis draw attention to the need for decision-makers in Romania (Government, Ministry of Health) to implement health policies that support the allocation of investments towards financially vulnerable categories and the healthcare domain, in order to maximize the efficiency of medical systems.

The post-crisis period not only in Romania, but also worldwide is followed by the Coronavirus crisis, some of the immediate effects of the lockdown policies and government regulations being similar to those observed in an economic crisis [40,41]. The Coronavirus crisis in Romania has already created the premises of a new economic crisis (unemployment, economic sectors that are frozen), with all its associated parameters, significantly changing individual and social behavior. Applying political and budgetary measures along with lockdown policies can generate “unintended consequences” [42], both at a socioeconomic level (implicitly also on the healthcare system), and at an individual level, under the aspect of adaptation and responsibility towards imposed safety measures. Although the European Commission foresees an economic growth in the Euro area to follow the decreased economic-financial activity recorded in the second half of 2020, the possibility of a new pandemic wave in the first half of 2021, triggers in Romania, similarly to other countries, the necessity for imposing measures to limit the spread of the virus, that will reflect on the healthcare system and on public health. Regarding medical care, the World Health Organization Europe has already warned that the effect of the pandemic on the treatment and care of oncologic patients is “catastrophic” [43], with a third of member countries having interrupted oncologic services. The Coronavirus pandemic can be viewed as a double crisis, both medical and economic [44,45]. Due to the fact that the economic impact of the pandemic is uneven [46], the pace of recovery will vary significantly from country to country. Thus, starting with March 2020, in the context of the COVID-19 pandemic, restrictive measures began to be imposed in Romania, thus generating the setting of a new economic instability that seriously affects, among other sectors, the healthcare system [47]. In addition, the recent evolution of the pandemic in Romania is more severe than predicted in the first half of last year, the effects of this evolution are expected to be long-lasting. Moreover, it is estimated that the financial instabilities and restrictive measures will significantly increase the budgetary deficit in the following period [48]. Governmental policies have reduced economic activity by deciding to suspend the activity of certain sectors in order to limit the spread of coronavirus. Since the second half of last year, layoffs have been made, including in the lower-wage sectors of the economy [49,50]. 

EU member states have already taken political, budgetary, and liquidity measures to increase the capacity of their health systems, but also to provide support/assistance to severely affected people and sectors, by developing a conceptual framework for identifying health systems’ responses to the COVID-19/economic crisis and by establishing models of good practices in the field of health policies. The strain of this pandemic on the healthcare system is very likely to present with delayed effects on the prognosis of multiple diseases. Treatment of cancer patients has been delayed in most countries, giving rise to the need of establishing measures that can ensure uninterrupted oncologic care [51]. However, such measures may be difficult to implement for healthcare systems that are both lacking infrastructure and sufficient staff, as can be seen in low- and middle-income countries. Thus, in the context of the aforementioned aspects, the model of delayed cancer diagnosis observed in the previous economic crisis can serve as a predictive tool for the current medical and financial instability setting. 

As a closing remark, we highlight that in low- and middle-income countries the struggle for the early diagnosis of breast cancer is further burdened by the lack or poor quality of an evidence base that can guide the accomplishment of attaining an efficient strategy for breast cancer control, especially during times of economic and medical crises. 

## 5. Conclusions

Considering the limited data available in the literature regarding the impact of the economic crisis on the diagnosis and prognosis of breast cancer in Romania, the present study offers valuable data regarding the regional dynamic of breast cancer cases in the western region. Moreover, it was observed that during the 2007–2012 period of economic instability recorded in Romania, breast cancer patients were diagnosed predominantly at a later stage, with a significantly higher proportion in the population from a rural setting. However, the present study has several limitations that must be acknowledged. Firstly, presented data are the results of univariate analysis. Second, due to the limited parameters recorded through regional registries, not all variables that may explain the change in the moment of diagnosis could be included in the analysis. As future research directions, we consider that a regression modeling of potential factors influencing the stage at diagnosis would provide more statistically valid data. Furthermore, an evaluation of specific social factors that determine a change in behavior and addressability to health services is warranted. 

Ensuring and maintaining population health represent important aspects, particularly during periods of great socioeconomic stress, such as an economic crisis. Along with other causes, the economic factor may contribute to a delay in the moment of diagnosis of breast cancer. This principle can easily apply to other cancer types or diseases, determining more time-consuming and expensive management, generally with a poor long-term outcome. The health-associated impact of economic instability requires further study for the development of sustainable, preventive strategies for decreasing morbidity and mortality during periods of economic crisis. 

## Figures and Tables

**Table 1:** Distribution of breast cancer patients by study period.

Period	No. of Patients/Year (Mean ± SD)	Total No. of Patients (%)
pre-crisis	223.7 ± 15.7	1342 (27.2)
crisis	211.3 ± 22.2	1268 (25.7)
post-crisis	331.3 ± 70.6	2319 (47.0)

**Table 2:** Distribution of study group by the moment of diagnosis.

Period	No. of Patients with Early-Stage Breast Cancer (%)	No. of Patients with Late-Stage Breast Cancer (%)
pre-crisis	712 (53.1)	630 (46.9)
crisis	554 (43.7)	714 (56.3)
post-crisis	1291 (55.7)	1028 (44.3)

**Table 3:** Distribution of study group by setting and moment of diagnosis.

Period	No. of Patients from Urban Setting (%)		No. of Patients from Rural Setting (%)	
	Early *	Advanced **	Early *	Advanced **
pre-crisis	424 (56.4)	328 (43.6)	288 (48.8)	302 (51.2)
crisis	384 (49.2)	396 (50.8)	170 (34.8)	318 (65.2)
post-crisis	851 (59.8)	571 (40.2)	440 (49.1)	457 (50.9)

* early–breast cancer stages 0, I, and II; ** advanced–breast cancer stages III and IV.

## Data Availability

The data presented in this study are available on request from the corresponding author. The data are not publicly available due to privacy restrictions.
